# Acute mammary and liver transcriptome responses after an intramammary *Escherichia coli* lipopolysaccharide challenge in postpartal dairy cows

**DOI:** 10.14814/phy2.12388

**Published:** 2015-04-28

**Authors:** Andrea Minuti, Zheng Zhou, Daniel E Graugnard, Sandra L Rodriguez-Zas, Alejandro R Palladino, Felipe C Cardoso, Erminio Trevisi, Juan J Loor

**Affiliations:** 1 Istituto di Zootecnica, Facoltà di Scienze Agrarie, Alimentari e Ambientali, Università Cattolica del Sacro CuorePiacenza, Italy; 2 Department of Animal Sciences, Division of Nutritional Sciences, University of IllinoisUrbana, Illinois; 3 Facultad de Agronomia, Universidad de Buenos AiresBuenos Aires, Argentina

**Keywords:** Immune function, inflammation, mastitis, metabolism

## Abstract

The study investigated the effect of an intramammary lipopolysaccharide (LPS) challenge on the bovine mammary and liver transcriptome and its consequences on metabolic biomarkers and liver tissue composition. At 7 days of lactation, 7 cows served as controls (CTR) and 7 cows (LPS) received an intramammary *Escherichia coli* LPS challenge. The mammary and liver tissues for transcriptomic profiling were biopsied at 2.5 h from challenge. Liver composition was evaluated at 2.5 h and 7 days after challenge, and blood biomarkers were analyzed at 2, 3, 7 and 14 days from challenge. In mammary tissue, the LPS challenge resulted in 189 differentially expressed genes (DEG), with 20 down-regulated and 169 up-regulated. In liver tissue, there were 107 DEG in LPS compared with CTR with 42 down-regulated and 65 up-regulated. In mammary, bioinformatics analysis highlighted that LPS led to activation of NOD-like receptor signaling, Toll-like receptor signaling, RIG-I-like receptor signaling and apoptosis pathways. In liver, LPS resulted in an overall inhibition of fatty acid elongation in mitochondria and activation of the p53 signaling pathway. The LPS challenge induced changes in liver lipid composition, a systemic inflammation (rise of blood ceruloplasmin and bilirubin), and an increase in body fat mobilization. The data suggest that cells within the inflamed mammary gland respond by activating mechanisms of pathogen recognition. However, in the liver the response likely depends on mediators originating from the udder that affect liver functionality and specifically fatty acid metabolism (*β*-oxidation, ketogenesis, and lipoprotein synthesis).

## Introduction

The peripartal or “transition” period in taurine cattle is considered the most important phase of the lactation cycle because during this time animals are most-susceptible to metabolic or infectious disorders (Drackley 1999; Van Knegsel et al. 2014). The immune and innate host resistance mechanisms (Mallard et al. 1998) are impaired during the peripartal period, often rendering cows unable to mount an effective immune response (Goff and Horst 1997; Lacetera et al. 2005). As a consequence, relative to the rest of the lactation cycle (Mulligan and Doherty 2008), the transition period is when the highest proportion of intramammary infections occur (Oliver and Sordillo 1988; Oviedo-Boyso et al. 2007; Aitken et al. 2011).

The acute-phase response (APR) begins once a pathogen is detected by the receptors on the epithelial cells of the mammary gland, leading to activation of the immune system to eliminate the pathogen. Lipopolysaccharide (LPS) is a major component of the outer membrane of gram-negative bacteria; hence, an LPS challenge is commonly used to evaluate the effect of the APR on the animal's immune response. In mammalian species, LPS generates a local and systemic response involving immune cells and key organs (Hoeben et al. 2000; Mehrzad et al. 2001a), of which the liver is likely the most important. For instance, liver is responsible for maintaining the level of essential metabolites during critical stages of metabolic stress and synthesizes the necessary components for an immediate defense (acute-phase proteins, APP) at the site of tissue damage (Baumann and Gauldie 1994; Bertoni and Trevisi 2013).

The metabolic and hormonal changes associated with the onset of lactation affect immune function, not only of immune cells such as neutrophils but also in liver and in mammary gland (Hoeben et al. 2000; Goff 2006). Previous data indicated that use of an LPS challenge as an experimental tool generates a more pronounced response during early lactation (Lehtolainen et al. 2003), thus, it represents a suitable model for simulating the response that dairy cows infected with mastitis may undergo. As such, both local (mammary) and peripheral (liver) effects of the APR on the tissue transcriptome can be studied during a physiological stage (early post-partum) when cows are immuno-compromised.

The hypothesis was that an inflammatory challenge soon after parturition would alter both the mammary and liver transcriptome. Furthermore, some of the alterations in the liver transcriptome would be reflected at least in part by changes in hepatic lipid composition. The main objective of this study was to investigate simultaneously the acute response of the mammary and liver transcriptome resulting from a mammary inflammatory challenge soon after the onset of lactation. A second objective was to link transcriptome changes with tissue and blood biomarkers.

## Materials and Methods

### Animals, diets, and management

All procedures involving animals received approval from the University of Illinois Institutional Animal Care and Use Committee (IACUC, protocol #06145). The experimental design and management details have been published previously (Graugnard et al. 2013). Briefly, fourteen Holstein cows entering their second or greater lactation were used. Cows were housed in a ventilated tie-stall barn and were fed a common lactation diet (Net energy of lactation = 1.69 Mcal/kg DM) as a total mixed ration once daily (0600 h) and milked twice daily (0400 and 1600 h). The approach for calculation of energy balance was reported previously (Graugnard et al. 2012).

### Lipopolysaccharide challenge

At 7 days postpartum, seven of the 14 cows received an intramammary *E. coli* LPS challenge (200 *μ*g, strain 0111:B4, cat. # L2630, Sigma Aldrich, St. Louis, MO) dissolved in 20 mL of 0.09% sterile physiological saline (Hospira, Lake Forest, IL). Seven cows served as controls (CTR), and received the same volume of sterile saline only. Prior to LPS or control treatment (∼2 days), fore-milk samples from all quarters of each cow were cultured and confirmed to be bacteriologically negative. Cows received their respective treatments immediately after the AM milking (0530 h). One rear mammary quarter was disinfected with cotton wool presoaked in 70% ethanol and the LPS or CTR solution were infused via a sterile disposable syringe fitted with a sterile teat cannula using the full-insertion infusion method. The quarter was thoroughly massaged.

### Mammary and liver biopsies

The day of the challenge (7 days postpartum), simultaneous biopsies of mammary and liver were harvested. The biopsy procedures were performed at 0730 h, approximatively 2.5 h after the intramammary LPS or CTR challenge to avoid excessive infiltration of polymorphonuclear neutrophils (PMN), that is, as a means to avoid excessive confounding on tissue gene expression. Biopsies of mammary tissue were collected from a rear quarter in all the cows according to procedures described elsewhere (Bionaz and Loor 2007). The samples (∼1 g) were frozen immediately in liquid nitrogen and stored at −80°C until RNA extraction. Biopsies of liver were harvested on the day of the challenge immediately before the mammary biopsies (2.5 h after challenge), and then 7 days after the challenge (14 days postpartum). Samples were collected via puncture biopsy under local anesthesia as previously reported (Loor et al. 2007). Liver tissue (∼1 g) was frozen immediately in liquid nitrogen and stored at −80°C until RNA extraction. A subsample of liver tissue was used for analysis of total lipids and triacylglycerol (TAG) (Loor et al. 2005, 2006). Cows were monitored for 2 weeks after biopsy in order to evacuate blood clots and ensure proper healing of the incision (cleaning and iodine ointment applications were performed when necessary).

### RNA extraction, microarrays, and quantitative PCR

RNA was extracted from mammary and liver tissue using established protocols (Mehrzad et al. 2001a). Transcript profiling of mammary and liver was with a bovine oligonucleotide (70-m) microarray with >13,000 annotated sequences developed at the University of Illinois (Loor et al. 2007). Details on the development, annotation, use of this microarray, and methods for microarray hybridization and scanning have been reported previously (Loor et al. 2007). In addition to the microarray, liver RNA was used to measure 12 target genes involved in metabolism, inflammation, and cellular stress using quantitative RT-PCR (Graugnard et al. 2013).

### Blood samples and analyses

Blood samples were collected at −2, 3, 7, and 14 days from the LPS challenge. Samples were analyzed for metabolic biomarkers [glucose (mmol/L), total cholesterol (mmol/L), nonesterified fatty acids or NEFA (mmol/L), *β*-hydroxybutyrate or BHBA (mmol/L), creatinine (mmol/L), urea (mmol/L), aspartate aminotransferase or GOT (U/L)], and oxidative-inflammatory biomarkers [albumin (g/L), total bilirubin (*μ*mol/L), haptoglobin (g/L), ceruloplasmin (*μ*mol/L), reactive oxygen metabolites or ROM (mg H_2_O_2_/100 mL)] and plasma vitamin A, vitamin E, and *β*-carotene. Details of sampling procedures and analyses have been published (Graugnard et al. 2013).

### Statistical analyses

Data from microarrays were normalized for dye and microarray effects (i.e., Lowess normalization and microarray centering) and used for statistical analysis as described previously (Loor et al. 2005, 2006, 2007). Data were analyzed using the Proc MIXED procedure of SAS (SAS Inst. Inc., Cary, NC). Fixed effects were treatment (LPS, not-LPS) and dye (Cy3, Cy5). Random effects included cow and microarray. Raw *P* values for the treatment effect were adjusted using Benjamini and Hochberg's false discovery rate (FDR). Differentially expressed genes (DEG) between LPS and CTR were considered significant at an FDR-adjusted *P *≤* *0.05 for treatment. For a more stringent characterization between the two treatments, a 1.5-fold difference in mRNA expression was set as threshold among DEG. Quantitative RT-PCR, liver composition and blood data were tested for normal distribution using the Shapiro–Wilk test (SAS Inst. Inc.) and normalized by natural log-transformation when necessary. The temporal data were analyzed using MIXED procedures of SAS (SAS Inst. Inc.; release 8.0) with repeated measures. The statistical model included treatment (CTR or LPS), time (days from treatment), and interaction of treatment × time as fixed effects; cow was the random effect. All values are presented as the means and standard error of the mean (SEM). All means were compared using the PDIFF statement of SAS and statistical significance was declared at a *P*-value ≤ 0.05 or 0.01. The microarray data files are available at the NCBI GEO database (series GSE64225).

### Microarray data mining

Data mining was performed using the dynamic impact approach (DIA) (Bionaz et al. 2012) and ingenuity pathway analysis (IPA; Ingenuity Systems, Inc., http://www.ingenuity.com). The IPA was used to study the effect of intramammary LPS challenge on transcription regulators and target genes among the DEG. In addition, IPA network analysis was used to evaluate the possible effect of intramammary LPS challenge on the liver transcriptome. To perform that analysis, the DEG from mammary tissue classified by IPA as cytokines were used to create networks with transcription regulators in the liver dataset.

## Results

### Mammary transcriptome

**Figure 1:**
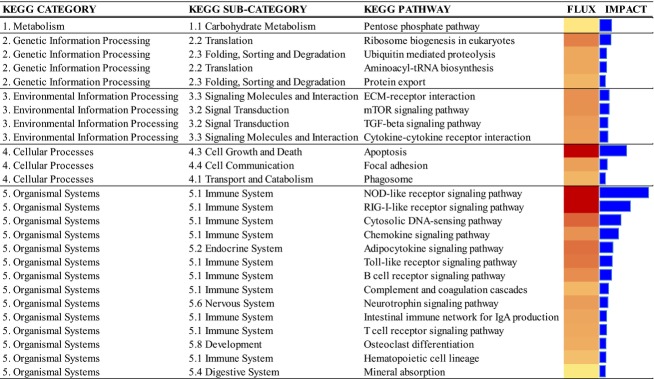
Results of flux and impact analysis of KEGG pathways using the dynamic impact approach (DIA) and the mammary differentially expressed genes after intra-mammary LPS challenge in early lactation (7 days) Holstein cows.

A total of 189 transcripts were differentially expressed in the LPS compared with CTR group. Among these, 20 DEG were down-regulated and 169 up-regulated, and the fold-changes ranged between 6.55 and −2.62. The DIA analysis highlighted as the most-impacted and activated by LPS the KEGG pathways NOD-like receptor signaling pathway (NLRs), RIG-I-like receptor signaling pathway (RLRs), apoptosis, cytosolic DNA-sensing pathway, chemokine signaling pathway, adipocytokine signaling pathway and toll-like receptor signaling pathway (TLR) (Fig.1). The IPA analysis also underscored the presence of 13 transcription regulators among the 189 DEG (Table1). Two were down-regulated and 11 were up-regulated.

**Table 1:** Transcription regulators and their respective target genes among the differentially expressed genes in mammary tissue detected at 2.5 h from an LPS intra-mammary challenge at 7 days postpartum in Holstein cows. The fold-change response between LPS and control for each gene is reported in parenthesis.

Transcription regulators	*n*	Target gene
*MYC* (1.53)	57	*BCL2A1* (4.74), *NFKBIA* (4.28), *SOD2* (3.43), *CD69* (3.34), *SLC2A3* (3.23), *THBS1* (2.97), *NOP58* (2.82), *DDX3X* (2.71), *SDCBP* (2.57), *GNL3* (2.39), *CXCL8* (2.33), *TOP1* (2.31), *CD9* (2.28), *EIF4E* (2.24), *RSAD2* (2.13), *TIMP1* (2.10), *PLAUR* (2.08), *CEBPD* (2.08), *DDX5* (2.04), *IL6* (2.03), *PMP22* (2.03), *OAS1* (2.02), *CFLAR* (1.96), *VCAM1* (1.89), *KLF5* (1.86), *THBS2* (1.84), *PLAT* (1.80), *HSP90AA1* (1.72), *PTMA* (1.71), *FTH1* (1.70), *PIM2* (1.70), *PSMA2* (1.70), *EIF4A1* (1.69), *PTGS2* (1.68), *RHOB* (1.67), *IFRD1* (1.67), *PLAU* (1.63), *MCL1* (1.62), *ACTB* (1.60), *HIF1A* (1.59), *STAT3* (1.59), *IRF1* (1.58), *ICAM1* (1.57), *NOS2* (1.57), *ZFP36* (1.57), *IKBKB* (1.57), *GADD45A* (1.55), *LIF* (1.55), *GCSH* (1.53), *COL4A1* (1.53), *PTPN11* (1.50), *PLSCR1* (1.51), *CAPZB* (1.511), *CD40* (1.50), *TSLP* (1.50), *YY1* (−1.54), *RARRES1* (−1.57)
*NFKBIA* (4.28)	45	*S100A9* (6.55), *BIRC3* (5.73), *BCL2A1* (4.74), *SOD2* (3.43), *CCL2* (3.36), *CD69* (3.34), *CCL20* (2.98), *IL1RN* (2.91), *CXCL2* (2.89), *DDX3X* (2.71), *CXCL8* (2.33), *FSCN1* (2.31), *EIF4E* (2.24), *ERRFI1* (2.22), *TNFAIP2* (2.17), *TIMP1* (2.10), *BCL3* (2.10), *CEBPD* (2.08), *IL6* (2.03), *CFLAR* (1.96), *VCAM1* (1.89), *HSPA8* (1.87), *KLF5* (1.86), *CD80* (1.81), *PLAT* (1.80), *CASP4* (1.76), *RIPK2* (1.72), *RPS6KA3* (1.71), *PSMA2* (1.70), *PTGS2* (1.68), *RHOB* (1.67), *PLAU* (1.63), *IFI6* (1.60), *HIF1A* (1.59), *STAT3* (1.59), *IRF1* (1.58), *ICAM1* (1.57), *NOS2* (1.57), *IKBKB* (1.57), *GADD45A* (1.55), *LIF* (1.55), *MYC* (1.53), *PTPN11* (1.51), *TIMP3* (1.51), *CD40* (1.50)
*STAT3 (1.59)*	42	*NFKBIA (4.28), SOD2 (3.43), CCL2 (3.36), CCL20 (2.98), THBS1 (2.97), IL1RN (2.91), CXCL2 (2.89), GNL3 (2.39), CXCL8 (2.33), FSCN1 (2.31), CD9 (2.28), ERRFI1 (2.22), TIMP1 (2.10), BCL3 (2.10), PLAUR (2.08), CEBPD (2.08), IL6 (2.03), OAS1 (2.02), CFLAR (1.96), CD80 (1.81), CASP4 (1.76), HSP90AA1 (1.72), PIM2 (2.03), PTGS2 (1.68), PLAU (1.63), MCL1 (1.62), IFI6 (1.60), HIF1A (1.59), CASP7 (1.59), IRF1 (1.58), ICAM1 (1.57), NOS2 (1.57), ZFP36 (1.57), IKBKB (1.57), GADD45A (1.55), LIF (1.55), MYC (1.53), PTPN11 (1.51), TFPI2 (1.51), PLSCR1 (1.51), CD40 (1.50), TSLP (1.50)*
*HIF1A* (1.59)	28	*NFKBIA* (4.28), *SLC2A3* (3.23), *CXCL8* (2.33), *FSCN1* (2.31), *TOP1* (2.31), *EIF4E* (2.24), *PLAUR* (2.08), *IGFBP3* (2.05), *DDX5* (2.04), *IL6* (2.03), *HSPA8* (1.87), *KLF5* (1.86), *HSP90AA1* (1.72), *PIM2* (1.70), *PTGS2* (1.68), *MCL1* (1.62), *ACTB* (1.60), *STAT3* (1.5), *DNAJB1* (1.59), *IRF1* (1.58), *NOS2* (1.57), *ZFP36* (1.57), *IKBKB* (1.57), *LIF* (1.55), *MYC* (1.53), *COL4A1* (1.53), *YY1* (−1.54), *SLC40A1* (−1.54)
*IRF1* (1.58)	22	*NFKBIA* (4.28), *CCL2* (3.36), *IL1RN* (2.91), *CXCL8* (2.33), *TNFAIP2* (2.17), *RSAD2* (2.13), *BCL3* (2.10), *IL6* (2.03), *FGL2* (2.02), *OAS1* (2.02), *VCAM1* (1.89), *IFIT2* (1.87), *HSP90AA1* (1.72), *PTGS2* (1.68), *HIF1A* (1.59), *STAT3* (1.59), *CASP7* (1.59), *NOS2* (1.57), *IKBKB* (1.57), *LIF* (1.55), *MYC* (1.53), *CD40* (1.50)
*CEBPD (2.08)*	11	*BCL2A1 (4.74), NFKBIA (4.28), CCL20 (2.98), CXCL8 (2.33), IL6 (2.03), PTGS2 (1.68), STAT3 (1.59), NOS2 (1.57), IKBKB (1.57), LIF (1.55), MYC (1.53)*
*ZFP36* (1.57)	10	*BIRC3* (5.74), *CXCL8* (2.33), *IL6* (2.03), *PTGS2* (1.68), *HIF1A* (1.59), *STAT3* (1.59), *LIF* (1.55), *EXOSC9* (1.54), *GABPB1* (1.54), *MYC* (1.53)
*YY1* (−1.54)	10	*DDX3X* (2.71), *DDX5* (2.04), *ACTB* (1.60), *HIF1A* (1.59), *CASP7* (1.59), *GABPB1* (1.54), *MYC* (1.53), *TSLP* (1.50), *RAB8B* (1.50), *CR2* (−1.51)
*BCL3* (2.10)	10	*NFKBIA* (4.28), *CD69* (3.34), *CXCL8* (2.33), *PLAUR* (2.08), *IL6* (2.03), *CD80* (1.81), *STAT3* (1.59), *IRF1* (1.58), *ICAM1* (1.57), *CD40* (1.50)
*EHF* (−1.55)	7	*S100A9* (6.55), *S100A12* (5.71), *CCL20* (2.98), *IL1RN* (2.91), *TIMP1* (2.10), *PLAUR* (2.08), *PLAT* (1.80)
*CREM* (1.64)	7	*THBS1* (2.97), *CXCL8* (2.33), *ERRFI1* (2.22), *KLF5* (1.86), *RHOB* (1.67), *MCL1* (1.62), *NOS2* (1.57)
*KLF5* (1.86)	6	*S100A9* (6.55), *NFKBIA* (4.30), *CREM* (1.64), *HIF1A* (1.59), *IKBKB* (1.57), *MYC* (1.53)
*GABPB1* (1.54)	2	*ZFP36* (1.57), *YY1* (−1.54)

### Liver transcriptome

**Figure 2:**
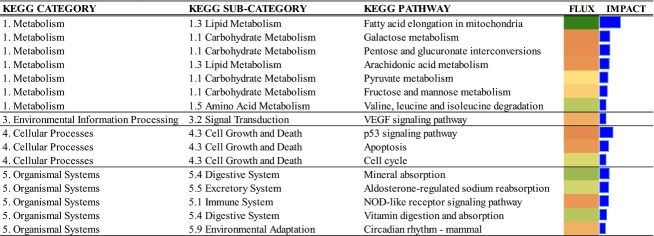
Results of flux and impact analysis of KEGG pathways using the dynamic impact approach (DIA) with the liver differentially expressed genes after intra-mammary LPS challenge in early lactation (7 days) Holstein cows.

The gene expression profiles revealed a total of 107 DEG. Among these 42 DEG were down-regulated and 65 were up-regulated, and the fold-changes ranged between 3.71 and −2.34. The DIA analysis highlighted the inhibition of fatty acid elongation in mitochondria, and the activation of p53 signaling pathway (Fig.2). The IPA analysis underscored the presence of 12 transcription regulators among the 107 DEG (Table2). Three were down-regulated and 9 were up-regulated.

**Table 2:** Transcription regulators and their respective target genes among the differentially expressed genes in liver tissue detected at 2.5 h from an LPS intra-mammary challenge at 7 days postpartum in Holstein cows. The fold-change response between LPS and control for each gene is reported in parenthesis.

Transcription regulators	*n*	Target gene
*MYC* (1.81)	24	*GADD45A* (3.29), *DDX3X* (2.49), *RHOB* (2.44), *PTGS2* (2.13), *BCL2A1* (1.90), *CE*BPD (1.79), *ZFP36* (1.79), *KLF10* (1.70), *FABP4* (1.69), *GADD45B* (1.63), *GADD45G* (1.62), *MCL1* (1.61), *XBP1* (1.57), *HSP90AA1* (1.56), *RRS1* (1.55), *TOP1* (1.54), *MMP7* (1.53), *LIF* (1.51), *IL1A* (1.50), *AKAP8* (1.50), *CAST* (−1.51), *GATA6* (−1.58), *KAT2B* (−1.71), *SERPINF1* (−1.85)
*CEBPD* (1.79)	6	*PTGS2* (2.13), *BCL2A1* (1.90), *MYC* (1.81), *FABP4* (1.69), *MAP3K8* (1.65), *LIF* (1.51)
*CREM* (2.11)	6	*RHOB* (2.44), *TIPARP* (1.90), *BHLHE40* (1.74), *BTG2* (1.72), *GADD45B* (1.63), *MCL1* (1.61)
*XBP1* (1.57)	6	*CXCL2* (2.02), *MYC* (1.81), *PIK3R1* (1.80), *TOP1* (1.54), *LIF* (1.51), *PPIB* (−1.57)
*ZFP36* (1.79)	5	*BIRC3* (2.89), *PTGS2* (2.13), *MYC* (1.81), *LIF* (1.51), *CNOT7* (−1.57)
*GATA6* (−1.58)	3	*MYC* (1.81), *LIF* (1.51), *APOB* (−1.80)
*KAT2B* (−1.71)	3	*PTGS2* (2.13), *MYC* (1.81), *ACTN2* (1.54)
*KLF10* (1.70)	3	*MYC* (1.81), *LIF* (1.51), *SIAH1* (−1.56)
*BHLHE40* (1.74)	2	*CREM* (2.11), *NEDD9* (1.77)
*BTG2* (1.72)	2	*CREM* (2.11), *NEDD9* (1.77)
*CNOT7* (−1.57)	2	*BTG2* (1.72), *ZFP36* (1.79)
*ACTN2* (1.54)	1	*KAT2B* (−1.71)

**Figure 3:**
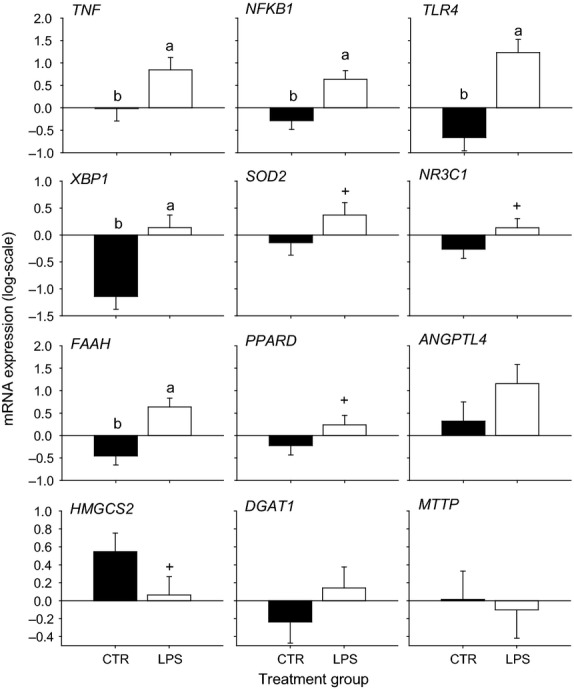
The effect of intra-mammary LPS or saline (CTR) challenge at 7 days postpartum in Holstein cows on mRNA expression of pro-inflammatory (*TNF*, *NFKB1*, *TLR4*), intracellular stress (*XBP1*, *SOD2*, *NR3C1*), PPAR signaling (*FAAH*, *PPARD*, *ANGPTL4*), and lipid metabolism (*HMGCS2*, *DGAT1*, *MTTP*) in liver tissue 2.5 h after intra-mammary LPS challenge. ^a–b^Denote differences due to LPS challenge. ^+^Denote tendencies (*P *<* *0.10) for differences due to LPS challenge.

Most of the genes evaluated by qPCR (Fig.3) were affected by the intramammary LPS challenge. Among these, we observed a marked up-regulation (*P *<* *0.05) of pro-inflammatory and stress-related genes. In addition, the LPS challenge resulted in the up-regulation of several PPAR signaling genes. However, the expression of the PPAR*α* target *HMGCS2*, which encodes the ketogenic rate-limiting enzyme, was down-regulated by LPS.

### Putative interactions between mammary and liver transcriptomes

**Figure 4:**
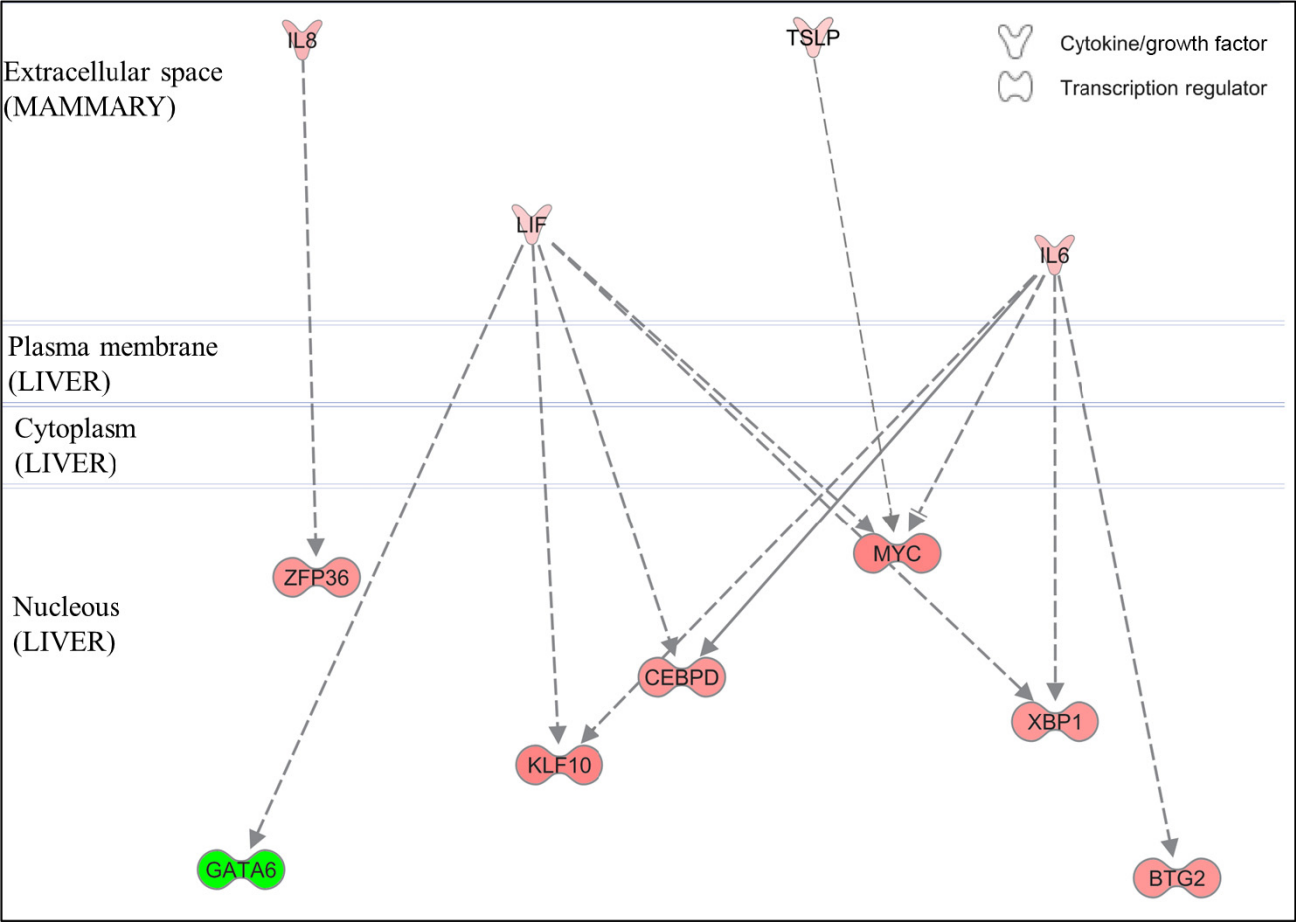
Networks among cytokines differentially expressed in the mammary gland and transcription regulators differentially expressed in liver at 2.5 h after intra-mammary LPS challenge. Figure generated by IPA. Red color denotes upregulation due to LPS and green downregulation due to LPS.

A direct effect of intramammary LPS challenge on the liver transcriptome was investigated using IPA software by mining for published relationships between the cytokines enriched in the mammary DEG dataset and the transcription regulators enriched in the liver DEG dataset. Four molecules classified as cytokines were upregulated in the mammary transcriptome (*IL6*, *IL8*, *LIF,* and *TSLP*) and were linked with seven transcription regulators in the liver (*ZFP36*, *GATA6*, *KLF10*, *CEBPD*, *MYC*, *XBP1,* and *BTG2*) (Fig.4).

### Effect of intramammary LPS challenge on plasma biomarkers

The intramammary LPS challenge resulted in few statistical effects on the inflammometabolic profiles analyzed. The pattern of changes of the main plasma biomarkers during the intramammary LPS challenge are reported in Table3. Haptoglobin, one of the most important positive APP, did not differ (*P *>* *0.10) between groups. Conversely, the LPS challenge increased the concentration of ceruloplasmin (*P *<* *0.05) and delayed the decrease in bilirubin (*P *<* *0.05) for 2 weeks in comparison with CTR. The concentration of ROM also increased after the LPS challenge, but compared with CTR the difference was less prolonged and with a tendency (*P *<* *0.10) to be higher 7 days after the LPS challenge. The plasma concentration of GOT was greater (*P *< 0.05) in the LPS group after parturition (and before the LPS challenge) but compared with CTR the reduction after the LPS challenge in the subsequent 2 weeks was more marked (*P *<* *0.06, group × time). The concentration of NEFA had an overall effect of treatment (*P *=* *0.02) due to an increase in concentration in LPS-challenged cows. No statistical effects were observed for BHBA concentration.

**Table 3:** The effect of intra-mammary LPS or saline (CTR) challenge at 7 days postpartum in Holstein cows on inflammometabolic blood biomarkers at −2, 3, 7 and 14 days from challenge.

Item	Group	Days from LPS challenge				SEM	*P*-value	
		−2	3	7	14		Group	Group × Time
Haptoglobin, g/L	CTR	0.78	0.73	0.39	0.40	0.13	0.94	0.01
	LPS	0.68	0.79	0.47	0.38			
Ceruloplasmin, µmol/L	CTR	3.17	3.28	3.24	3.39	0.23	0.02	0.75
	LPS	3.36	3.62	3.69^*^	3.52			
Bilirubin, µmol/L	CTR	4.06	2.97	2.55	2.02	0.78	0.05	0.03
	LPS	4.20	3.66	4.16^*^	2.49			
ROM, mgH_2_O_2_/100 mL	CTR	14.3	15.2	14.7	15.4	0.9	0.14	0.37
	LPS	14.6	16.3	16.3^+^	15.2			
GOT, U/L	CTR	116.8	117.3	107.7	86.9	16.1	0.28	0.06
	LPS	135.6	122.5	114.8	90.6			
NEFA, μEq/L	CTR	0.460	0.321	0.320	0.288	0.060	0.02	0.39
	LPS	0.466	0.509	0.457	0.355			
BHBA, mmol/L	CTR	0.527	0.663	0.625	0.688	0.08	0.74	0.15
	LPS	0.710	0.518	0.550	0.632			

SEM, standard error of means; ROM, reactive oxygen metabolites; GOT, aspartate aminotransferase; NEFA, non-esterified fatty acids; BHBA, β-hydroxybutyrate.

Statistical difference between groups at the same days was established by using a conventional *P* value lower of 0.1 (+) or 0.05 (^*^).

### Metabolism

**Figure 5:**
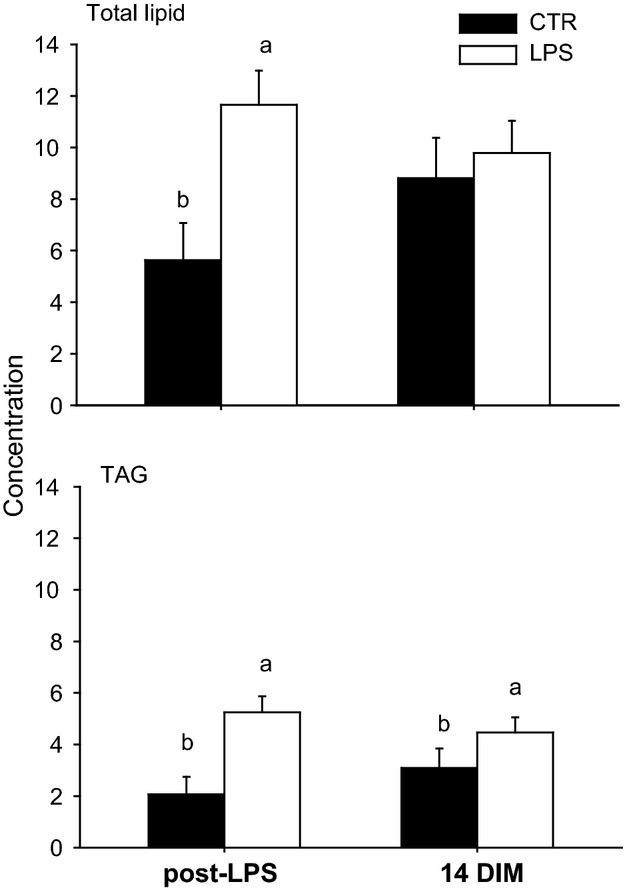
The effect of intra-mammary LPS or saline (CTR) challenge at 7 days postpartum in Holstein cows on liver lipid and triacylglycerol (TAG) concentration after LPS challenge and 14 days in milk (DIM). ^a-b^Denote differences (Group × time) due to LPS challenge.

The concentration of total lipid and TAG at 2.5 h postchallenge was greater (*P *<* *0.05) in the LPS compared with CTR group (Fig.5). The TAG concentration remained greater (*P *<* *0.05) in the LPS group at 7 days postchallenge. Although no statistically significant differences were detected for measures of metabolism and performance, there was a numerically lower response in dry matter intake for cows receiving the LPS challenge (Table4). Such response did not alter milk synthesis during the first 2 weeks of lactation.

**Table 4:** The effect of intra-mammary LPS or saline (CTR) challenge at 7 days postpartum in Holstein cows on dry matter intake, milk production, and energy balance.

Item	Group		SEM	*P*-value	
	CTR	LPS		Group	Group × time
DMI, day 7 to 14					
kg/day	18.5	15.9	1.5	0.18	0.47
% of body weight	2.67	2.20	0.26	0.16	0.61
Milk (kg/day), day 7 to 14	36.4	35.1	3.5	0.77	0.77
Energy balance, weeks 1 and 2					
Mcal/day	−6.5	−9.1	2.6	0.46	0.48
% of requirements	82.3	74.4	6.7	0.37	0.19

SEM, standard error of means.

Time effect was significant (*P *<* *0.05) for all items except milk production (*P *=* *0.55).

## Discussion

Two aspects are crucial to appreciate the novelty of this experiment utilizing LPS from *E. coli* 0111:B4 to mimic a mammary infection, (1) the transcriptome was analyzed in parallel in mammary and hepatic tissue; (2) the biopsy was carried out just 2.5 h after infection, thus, helping evaluate the early/initial/APR to inflammation. In fact, this time was selected in order to avoid excessive infiltration of PMN and the potential confounding on tissue gene expression. Furthermore, the LPS challenge was performed during the early postpartum or “fresh” period, which is a critical phase of lactation during which metabolic and infectious disorders are most common. Compared with latter stages of lactation, during this physiological stage, there is a lower number of circulating PMN which are immature, are functionally weak, and diapedesis into the infected quarters also is impaired (Mehrzad et al. 2001b).

### Intra-mammary LPS challenge alters the mammary transcriptome

The fact that LPS resulted in ∼200 DEG within 2.5 h agrees with Lehtolainen et al. (2003) who observed an increase in milk somatic cell count (a local response to infection) and Hoeben et al. (2000) who detected an increase in body temperature (systemic response to infection) within 2–6 h after an LPS challenge. The KEGG sub-categories with an activated flux are primarily related to the immune system and the various cellular systems that detect pathogens, that is, the pattern recognition receptors (PRR) used to detect signals originating from pathogens (PAMPs). The coordinated activation of the various PRR pathways induces multiple pro-inflammatory signaling pathways associated with the innate-immune response to mount an effective bactericidal response (Kawai and Akira 2009).

Activation of the various PRR converges on nuclear factor-kappa B (NF-kB), which is a key player in controlling both innate and adaptive immunity. Data from nonruminant models indicated that activation of NF-kB (via LPS and pro-inflammatory cytokines) and nuclear factor kappa-B kinase (including *IKBKB*) leads to phosphorylation of *NFKBIA* on the amino terminus at serine residues (Chen et al. 1995) resulting in its degradation, and allowing activated NF-kB to translocate to the nucleus. In this study, the up-regulation of both the enzyme inhibitor of nuclear factor kappa-B kinase subunit beta (*IKBKB*; fold change 1.6 in LPS vs. CTR) and the nuclear factor of kappa light polypeptide gene enhancer B-cells inhibitor-alpha (*NFKBIA*; fold change 4.3 in LPS vs. CTR) after LPS challenge supports the existence in bovine mammary of a negative feedback mechanism whereby activation of NF-kB induces its own inhibitor (*NFKBIA*) (Jobin and Sartor 2000).

The activation of the chemokine signaling pathway (including *CCL2*, *CCL20* and *CXCL2*), confirmed the initiation of the inflammatory immune response. Chemokines are small chemo-attractant peptides that act to guide leukocyte migration towards the site of infection (Oviedo-Boyso et al. 2007; Aitken et al. 2011). Using mammary epithelial cells, Gilbert et al. (2013) reported the existence of a core innate immune response partly shared by LPS and *S. aureus* including pro-inflammatory cytokines and chemokines (*IL6*, *IL8*, *CCL2*, *CCL20,* and *CXCL2*) and other inflammation-related genes (*NFKBIA*, *CEBPD,* and *PLAU*). Data from this study agree with those findings. It is noteworthy that Gilbert et al. (2013) detected a faster response and of a greater magnitude with *E. coli* than *S. aureus*. The main difference between both models was the activation of the type 1 interferon pathway by *E. coli*, which our data partly confirmed (Table1).

The upregulation of the genes *CFLAR*, *CASP7*, and *CASP4* following the LPS challenge was responsible for the significant activation of the KEGG pathway Apoptosis. In addition, the upregulation of *MYC*, *STAT3*, *IL6*, and *LIF* likely contributed to acute regulation of the balance between cell survival or death soon after the LPS challenge. The apoptosis pathway is a genetically controlled process of programmed cell death involved in the regulation of tissue homeostasis. Previous work revealed that mammary *E. coli* infection or a challenge of mammary cell cultures with LPS-induced cell death via apoptosis (Long et al. 2001; Baldi et al. 2012; Gilbert et al. 2013). Intramammary LPS challenge also increased expression of *CASP3* and *CASP7* in mammary tissue within 3 h of challenge (Bruckmaier 2005). The upregulation of *PLAU* and *PLAT* in this study, mediators of extracellular proteolytic events, also support the pro-apoptotic effect of LPS. Unlike a mastitis pathogen, this acute activation of apoptosis would not be expected to cause an impairment of milk synthesis (i.e., there was no difference in daily milk production) because the LPS effect is transient.

### Intramammary LPS challenge and the liver transcriptome

The production of pro-inflammatory cytokines (IL-1*β*, IL-6 and TNF*α*) upon LPS sets-off a systemic inflammatory response (Rinaldi et al. 2010), for which the liver plays a central role (Waldron et al. 2003a; Jiang et al. 2008). However, it has been argued that LPS does not enter into the circulation. Hence, the systemic effects after LPS or *E. coli* are due to pro-inflammatory cytokines which increase in blood (Hoeben et al. 2000). The pro-inflammatory cytokines stimulate the hepatocyte to activate the expression of genes to synthesize APP (e.g., ceruloplasmin, serum amyloid A, haptoglobin), a response confirmed by the data from this study. In fact, while in the mammary tissue we detected a direct effect of LPS on immune system pathways, in the liver we detected the involvement of systems of adaptation (pro-inflammatory, lipid metabolism and intracellular stress systems) probably as a response to signals originating from mammary gland. Overall, the liver PCR data demonstrating upregulation of pro-inflammatory and stress-related genes (Fig.3) agree with previous studies with midlactation cows underscoring a robust response of liver to an intramammary inflammatory challenge (Jiang et al. 2008; Vels et al. 2009; Jørgensen et al. 2012).

The observed inhibition of Fatty acid elongation in mitochondria after LPS because of the downregulation (fold change of −1.55 in LPS vs. control) of *ACAA2* along with downregulation of *HMGCS2* and *APOB* confirm that the APR regulates energy metabolism and lipoprotein synthesis in hepatocytes. The ACAA2 enzyme catalyzes the last step of mitochondrial fatty acid *β*-oxidation leading to the release of acetyl-CoA. The HMGCS2 enzyme catalyzes the rate-limiting step of ketogenesis and its level is strongly correlated with ketogenic rate and availability of acetyl-CoA (Hegardt 1999). Transcription of *APOB* is essential for synthesis of mature very-low density lipoproteins. Although other control mechanisms regulating ACAA2 besides transcription were not evaluated in this study, the pro-inflammatory cytokines and cortisol (Waldron et al. 2003a) released after LPS elicit a marked increase in NEFA uptake by liver (Khovidhunkit et al. 2004). If the capacity of liver to oxidize long-chain fatty acids in LPS-challenged cows was diminished by the down-regulation of *ACAA2*, then it would explain the greater tissue concentration of TAG (Fig.5) and lower lipoprotein export (Drackley 1999). The decrease in expression of *APOB* in the LPS group (fold change −1.8 in LPS vs. CTR) also supports this idea.

The activation of the p53 signaling pathway is induced by a number of stress signals, all impacting cellular homeostatic mechanisms that monitor and control the fidelity of DNA replication (Levine et al. 2006). The p53 protein, acting through suppression of NF-*κ*B, plays a “buffering” role for the innate immune responses in vivo as a negative regulator of inflammation (Gudkov et al. 2011). The activation of this pathway in response to genotoxic stress can block cell proliferation or induce cell death pathways (Kim et al. 2009). These are mechanisms to prevent DNA damage and allow cells time for DNA repair (Haanen and Vermes 1995). Overall, the upregulation of *TP53*, *MYC*, and *BCL2A1* (all play an important role in the control of apoptosis) (Askew et al. 1991; Vogler 2012) suggests a quick response by liver to maintain homeostasis.

The hepatic APR after an inflammatory challenge includes the production and secretion into the plasma of positive APP (Eckersall et al. 2006). Vels et al. (2009) using peak-lactation cows and a similar intramammary inflammatory challenge (200 *μ*g of *E. coli* LPS strain 0111:B4), detected a concentration of haptoglobin of ∼1.5 g/L after 3 days of the challenge. Grönlund et al. (2003) using *S. aureus* as intramammary challenge in mid-lactating cows detected ∼1.5 g/L of serum haptoglobin at 3–5 days after challenge, and Eckersall et al. (2006) reported that after induction of subclinical mastitis with *S. aureus* the haptoglobin concentration increased in serum from <0.01 to ∼0.6 g/L. In our study, the haptoglobin concentrations 2 days before the intramammary LPS challenge already were higher in the CTR than LPS group, and even higher than those reported by Eckersall et al. (2006). Thus, it is likely that at early stages of lactation (e.g., during the “fresh” period), when the haptoglobin concentrations often reach these values (Bionaz et al. 2007; Trevisi et al. 2012), the effect of subclinical mastitis could be masked.

Ceruloplasmin, another positive-APP involved in the synthesis of cytochrome oxidase and in antioxidant defenses (Eckersall and Conner 1988; Ceciliani et al. 2002), had the highest concentration in blood after the LPS challenge suggesting extrahepatic synthesis due to inflammation (Ceciliani et al. 2002). Conner et al. (1986) reported higher concentrations of ceruloplasmin in cows with mastitis in comparison with healthy cows. In a previous study (Trevisi et al. 2012), cows with elevated concentrations of ceruloplasmin before parturition but without an increase in plasma haptoglobin, had the worse health conditions during early lactation. Thus, ceruloplasmin could be a more useful index than haptoglobin for detecting inflammation during the peripartal period and also to identify infections induced by bacterial LPS.

The increase in bilirubin concentration after the LPS challenge is suggestive of an impairment of hepatic function (i.e., reduced bilirubin clearance) as a consequence of inhibition of mRNA expression of key hepatic enzymes involved in bilirubin clearance (Assenat et al. 2004). The increase in bilirubin after the LPS challenge agrees with one of our previous studies utilizing an intramammary LPS challenge (Graugnard et al. 2013), and with previous studies where cows with more severe health problems around parturition had the highest increase in this biomarker coupled with a more severe impairment of liver function (Bertoni et al. 2008; Trevisi et al. 2012).

## Conclusions

The intramammary LPS challenge induced a quick and strong transcriptome response in both mammary and liver. In the mammary tissue the response was associated with the activation of pattern recognition receptors that drive recruitment of leukocytes to the site of infection. The liver responded to maintain homeostasis partly via mediators originating from mammary gland. These mediators induced an inflammatory response in liver that, judging by the accumulation of lipid, impacted negatively some aspects of lipid metabolism. The blood and liver tissue biomarkers strongly suggested an impairment of liver functionality. The metabolic consequences of uncontrolled inflammation can be particularly harmful during the early stages of lactation when there is a marked degree of body fat mobilization. Longer term effects of intramammary mastitis infection occurring soon after parturition are needed to better dissect the consequences of activation of apoptotic pathways in terms of milk synthesis.
